# Prevalence of Biofilm-Forming Non-Typhoidal *Salmonella* Across the Farm-to-Fork Continuum: A Systematic Review and Meta-Analysis

**DOI:** 10.3390/microorganisms14071584

**Published:** 2026-07-20

**Authors:** Asmita Shrestha, Smriti Shringi, Babafela Awosile, Devendra H. Shah

**Affiliations:** Texas Tech University School of Veterinary Medicine, 7671 Evans Drive, Amarillo, TX 79106, USA; asmshres@ttu.edu (A.S.); smriti.shringi@ttu.edu (S.S.); babafela.awosile@ttu.edu (B.A.)

**Keywords:** biofilm, *Salmonella*, source, serogroup

## Abstract

Non-typhoidal *Salmonella* (NTS) remains a major cause of foodborne illness worldwide, and its persistence along the food-production continuum poses a significant public health challenge. Biofilm formation is an adaptive mechanism that enhances NTS survival and persistence outside the primary animal reservoir, particularly under extra-host stress conditions in food and environmental settings. We hypothesized that true biofilm-positive NTS are less prevalent in animal reservoirs and relatively enriched in food-, environmental-, and human-associated sources along the farm-to-fork continuum, reflecting their increased likelihood of persisting in foods and contributing to human exposure. Systematic review and meta-analysis were conducted following PRISMA guidelines, identifying 88 eligible studies; 57 qualified for systematic review, and 47 and 35 qualified for source- and serogroup-based meta-analyses, respectively. Descriptive synthesis revealed substantial biological and methodological heterogeneity across studies. Proportion-based analysis showed that true biofilm-positive (TBP) prevalence was lowest among animal isolates (58.4%), increased in food (67.7%) and human isolates (73.1%), and was highest among environmental isolates (88.1%) (χ^2^ test, *p* < 0.001). In the source-based meta-analysis, the pooled TBP prevalence was 73.9% (95% CI: 58.4–85.06%). Meta-regression demonstrated that the predicted proportion of TBP NTS among food and human sources was significantly higher compared with the animal reservoir (food: *p* = 0.0002; human: *p* = 0.0005), whereas the difference between the environmental and animal reservoirs was not statistically significant (*p* = 0.075). These findings suggest that biofilm-forming NTS are enriched outside the primary animal reservoir under extra-host stress conditions. The results raise testable hypotheses regarding biofilm-mediated persistence and enrichment across the food-production continuum and support future longitudinal studies to evaluate its role in transmission and targeted sanitation strategies.

## 1. Introduction

Non-typhoidal *Salmonella enterica* (NTS) is among the leading causes of foodborne illness worldwide and remains a major public health concern in both developed and developing countries. Globally, NTS is estimated to cause approximately 93.8 million foodborne illnesses and 155,000 deaths annually [1]. In the United States alone, NTS is responsible for an estimated 1.35 million infections, 26,500 hospitalizations, and 420 deaths each year [2].

NTS is genetically and antigenically diverse, with more than 1500 known serovars distinguished using the Kaufmann–White classification scheme based on variations in somatic (O) and flagellar (H) antigens [3]. However, only a subset of NTS serovars are commonly associated with foodborne illness. From 1996–2024, more than 80% of reported NTS-associated illnesses were attributed to fewer than 50 clinically significant serovars transmitted through contaminated food, environmental exposure, or contact with infected animals [4]. Among these, *S*. Enteritidis and *S*. Typhimurium are consistently identified among the most common serovars associated with foodborne illnesses globally [5].

One of the major factors contributing to the dissemination of NTS throughout the food-production continuum is its ability to form and persist within the biofilms [6]. NTS biofilms are structured multicellular communities encased within a self-produced extracellular matrix composed primarily of curli fimbriae (amyloid fibers) and cellulose [6,7]. While not all NTS produce biofilms [8], NTS strains that form biofilms exhibit enhanced tolerance to environmental stressors, disinfectants, desiccation, and antimicrobial agents [6].

The biofilm-mediated NTS persistence in food processing and in foods is particularly relevant within the farm-to-fork continuum, where NTS originating from food-animal production systems may persist during processing and subsequently contaminate food products destined for human consumption, ultimately escalating public health risks. For instance, outbreaks of foodborne salmonellosis have been traced back to persistent biofilm-associated contamination by NTS within food-processing environments [9,10,11], underscoring the potential of these organisms to persist for prolonged periods and repeatedly contaminate food products. It is important to note that biofilm formation represents a persistence-associated phenotype that enhances environmental survival, stress tolerance, and transmission potential under adverse conditions, rather than increased invasiveness or virulence [12].

Given that biofilm formation promotes increased tolerance to sanitation and disinfection, thereby prolonging survival in food production environments [12], enhanced biosecurity measures are often needed to eradicate biofilm-forming NTS from such environments to reduce public health risks. However, biofilm testing is not routinely incorporated as a readout during outbreak investigations or surveillance programs due to the lack of rapid, reliable, and validated biofilm testing assays [8]. Furthermore, many independent studies examining biofilm formation in NTS are limited by relatively small sample sizes, restricted serovar diversity, or imbalanced ecological sources relevant to the food chain. These limitations hinder a comprehensive understanding of the relationships among biofilm formation, ecological source, persistence, and transmission risk across the food-production continuum.

Here, we address this knowledge gap by a systematic synthesis of the prevalence and distribution of biofilm-producing NTS across animal, food, environmental, and human sources, based on the scientific evidence from peer-reviewed literature. The objective of this study was to conduct a systematic review and meta-analysis of biofilm-producing NTS isolated throughout the farm-to-fork continuum. We hypothesized that strong biofilm-forming NTS are less prevalent in animal reservoirs and relatively enriched in food-, environmental-, and human-associated sources along the farm-to-fork continuum. Such a pattern would support the hypothesis that biofilm-producing NTS are more likely to persist within the food chain and contribute disproportionately to contamination and human exposure.

## 2. Materials and Methods

### 2.1. Literature Search Strategy and Study Selection

Systematic review and meta-analysis were conducted in accordance with the Preferred Reporting Items for Systematic Reviews and Meta-Analyses (PRISMA) guidelines [13]. The PRISMA checklist is provided as a Supplementary File S5. The protocol was retrospectively registered with OSF Registries during peer review (10.17605/OSF.IO/2694M). A comprehensive literature search was performed using three electronic databases: PubMed, Scopus, and Web of Science, from each database’s inception through 14 March 2024, with no restriction on publication year (Compiled as Supplementary file S1_Table S1). The search strategy included the keywords “*Salmonella*” AND “biofilm” within article titles, abstracts, and keywords. Only peer-reviewed articles published in English were considered. The restriction to English-language publications was applied because accurate extraction of methodological details required for biofilm phenotype recategorization, including assay conditions, inoculum preparation, and scoring criteria, was most reliably accomplished from English-language full texts.

**Figure 1:**
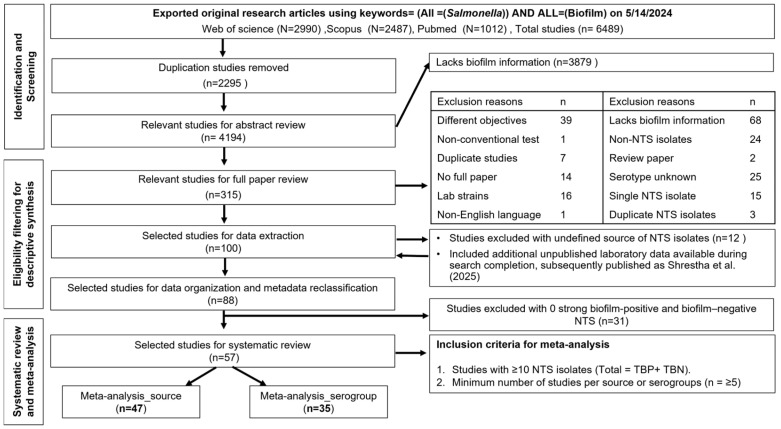
PRISMA flow diagram illustrates study identification, screening, eligibility assessment, and inclusion in the descriptive synthesis, systematic review, and meta-analysis.

Following the removal of duplicate records, titles and abstracts were screened for relevance (Supplementary file S1_Table S2). Full-text articles were subsequently assessed according to predefined eligibility and exclusion criteria (Section 2.2). The study selection process is summarized in Figure 1.

### 2.2. Eligibility and Exclusion Criteria

Studies (Supplementary file S1_Table S3) were included if they met the following criteria: (1) employed an observational study design; (2) evaluated biofilm formation in non-typhoidal *Salmonella enterica* isolates using at least one conventional biofilm assay, including the microtiter plate assay, Congo red-Coomassie brilliant blue (CRCBB) assay, calcofluor assay, or tube test; (3) were published in English in peer-reviewed journals; and (4) reported the serovar identity of the NTS isolates.

Studies were excluded if they: (1) were review articles, systematic reviews, or meta-analyses; (2) were conference proceedings or abstracts; (3) did not provide accessible full-text articles; (4) assessed biofilm formation using methods other than the predefined conventional assays; (5) were published in languages other than English; or (6) exclusively investigated host-adapted *Salmonella enterica* serovars, including *S.* Pullorum, *S.* Gallinarum, *S.* Typhi, *S.* Paratyphi, and *S.* Dublin.

For downstream analyses, isolates reported as unknown, non-typeable, or belonging to host-adapted serovars were excluded. Studies with source definitions that could not be clearly reassigned to one of the predefined ecological source categories were also excluded from source-based downstream analyses.

### 2.3. Data Extraction from the Eligible Studies for Descriptive Synthesis

Data from eligible studies were extracted and compiled using Microsoft Excel 365 (Microsoft Corporation, Redmond, WA, USA). The following variables were collected from each study: (1) country of study origin; (2) first author; (3) year of publication; (4) source of NTS isolation; (5) NTS serovar; (6) number of isolates tested; (7) biofilm assay used; (8) assay incubation temperature; (9) assay incubation duration; (10) microbiological medium used for biofilm assessment; and (11) reported biofilm phenotype. A descriptive synthesis of the complete dataset was conducted to characterize the biological and methodological structure. This descriptive synthesis included the distribution of serotypes, serogroups, biofilm assay methods, incubation temperatures, incubation durations, and ecological source categories. Graphical summaries were constructed to visualize the overall composition of the dataset and heterogeneity in both biological representation and experimental methodology before quantitative synthesis.

### 2.4. Source Recategorization for Systematic Review and Meta-Analysis

The source of NTS isolates is a primary moderator for our hypothesis testing by meta-analysis. Given that the studies reported diverse sources of NTS isolation, source definitions were reassigned into four standardized categories for downstream analysis as follows. This reclassification was performed to improve comparability across studies and to support ecological interpretation across the farm-to-fork continuum.

1. Animal: live animal sources, including poultry, pigs, cattle, reptiles, and wild animals;
2. Environment: farm environments, slaughterhouse environments, food-processing environments, feed-factory environments, and related non-host environmental sources;
3. Food: food-derived sources, including meat, vegetables, seafood, and other foods;
4. Human: clinical or fecal isolates derived from humans.

### 2.5. Biofilm Phenotype Recategorization for Systematic Review and Meta-Analysis

We recently reported that weak and moderate biofilm phenotypes frequently show assay-dependent or uncertain biofilm characteristics that may result in false-positive or false-negative inferences without further confirmation [8]. In line with our report, the biofilm phenotypes reported by studies included in our descriptive synthesis varied widely based on the biofilm tests, conditions of testing, and the phenotypic inferences. Because the biofilm phenotype of NTS isolates serves as a dependent variable for our hypothesis testing in this meta-analysis, the following criteria were adapted for reclassification of biofilm phenotypes [8]. Any isolate reported as a strong biofilm producer by any method (e.g., microtiter plate, tube, or CRCBB test) was classified as true biofilm-positive (TBP) (Supplementary file S1). Any isolate reported as negative by all methods applied within a study was classified as true biofilm-negative (TBN). In the CRCBB test, isolates with the reported phenotype of red dry and rough (*rdar*) colonies were classified as TBP, and isolates with the reported phenotype of smooth and white (*saw*) colonies were classified as biofilm-negative (TBN). Isolates reported as any other colony morphotype on the CRCBB test or reported as weak or moderate biofilm producers across all tests were excluded from downstream analyses.

### 2.6. Systematic Review of Eligible Studies

A proportion-based descriptive analysis was conducted using the dataset from 57 eligible studies to summarize TBP prevalence by ecological sources. For each study, the proportion of TBP isolates was calculated as the number of TBP isolates divided by the total number of isolates classified as TBP and TBN. For each source category, the corresponding 95% confidence intervals were calculated. An omnibus *chi*-square test of homogeneity was then used to assess whether TBP proportions differed significantly across source categories. Pairwise comparisons were subsequently conducted using two-proportion *z*-tests with Bonferroni correction to control the familywise type-I error rate. Because the included studies originated from diverse geographic regions and independent sampling frameworks, this analysis was intended to be descriptive and exploratory.

### 2.7. Meta-Analysis

The Excel dataset was imported into the R (*v* 4.5.3) statistical environment, and all quantitative analyses were conducted using the *metafor* package *v* 4.8.0 [14]. In each study, the logit-transformed proportion of TBP isolates was calculated as the primary effect size:

$$\mathrm{logit}(P)=log\left(\frac{x_{i}}{n_{i}-x_{i}}\right)$$

where $x_{i}$ represents the number of TBP isolates and $n_{i}$ represents the total number of isolates classified as TBP or TBN. The corresponding sampling variance was calculated as

$$v_{i}=\frac{1}{x_{i}}+\frac{1}{n_{i}-x_{i}}$$

To improve statistical reliability and reduce sparse subgroup estimates, quantitative meta-analysis included only source categories or serogroups represented by at least five independent studies and containing a minimum of 10 isolates per study-level observation. These stringent thresholds were selected to improve the stability and interpretability of pooled estimates as described previously [15].

Because substantial heterogeneity among studies was anticipated due to differences in assay methodology, incubation conditions, serovar composition, and isolate sources, random-effects meta-analysis models were used throughout the analysis [16]. To account for the hierarchical structure of the data, including multiple observations derived from the same publication, multilevel mixed-effects models were fitted using restricted maximum likelihood (REML) with study identifier included as a random effect.

### 2.8. Source and Serogroup-Level Meta-Regression

In this analysis, the proportion of TBP isolates was treated as the outcome variable, while ecological source and serogroup were evaluated as moderators to explain between-study variability in TBP prevalence using mixed-effects multilevel meta-regression models. The source of isolation was considered the primary moderator, given its direct relevance to the study’s ecological hypothesis regarding persistence across the farm-to-fork continuum. Serogroup was analyzed separately as a secondary, exploratory moderator to assess whether broad antigenic groupings were associated with TBP.

Baseline and moderator models were compared using likelihood-based fit statistics, including Akaike Information Criterion (AIC), Bayesian Information Criterion (BIC), deviance statistics, and log-likelihood [17]. The proportion of between-study variance explained by each moderator was quantified using pseudo-R-squared. The omnibus moderator test (QM) was used to assess the overall significance of each moderator, whereas residual heterogeneity following moderator inclusion was evaluated using QE statistics. Within each moderator model, pairwise comparisons between categories were performed using contrast vectors applied to the model coefficients, with standard error derived from the variance-covariance matrix. Statistical significance of pairwise contrasts was assessed using Wald *Z*-tests (*p* < 0.05).

### 2.9. Best Model Selection for Multivariable Meta-Analysis:

To determine whether the inclusion of moderators improved model fit beyond the baseline model (without the moderator), the heterogeneity measures I^2^, R^2^, and Tau^2^ of univariate and multivariate models were compared with those of the baseline models. Univariate meta-analysis was conducted using the same method as discussed above for source and serogroup in the 47 studies, with moderators (incubation temperature, incubation days, biofilm tests, source, and year) and binary indicators for serogroups D1, C1, C2-C3, and B1, one at a time. Two multilevel multivariate meta-analysis models: model 1 containing all those moderators and model 2 containing source and test (determined significant from the univariate meta-analysis). These two models were nested and differed in fixed-effect structure; both were refitted using maximum likelihood (ML) estimation rather than REML. Model fit was compared using a likelihood-ratio test, together with Akaike Information Criterion (AIC), Bayesian Information Criterion (BIC), and sample size-corrected AIC (AICc), with lower information criterion values indicating better fit.

### 2.10. Publication Bias Assessment

Publication bias was assessed for both the source-level and serogroup-level meta-analyses using Egger’s weighted regression test and Begg’s rank correlation test [18,19]. In addition, the trim-and-fill method was applied to estimate the number of potentially missing studies and to calculate bias-adjusted pooled estimates [20,21]. All publication bias analyses were assessed using a standard univariate random-effects model (.rma) function within the *metafor* package in R, with a significance threshold of p<0.05.

## 3. Results and Discussion

### 3.1. Study Selection and Descriptive Synthesis of the Dataset

PRISMA flow illustrating study identification, screening, eligibility assessment, and inclusion in the descriptive synthesis, systematic review, and meta-analysis is summarized in Figure 1. The data curated for descriptive synthesis, systematic review, and meta-analysis are summarized in Supplementary file S1 (Tables S1–S8). A total of 6489 articles (Supplementary file S1_Table S1) were initially identified through database searching. Following the removal of duplicate records, 4194 articles (Supplementary file S1_Table S2) were retained for the initial screening of titles and abstracts. Based on predefined eligibility criteria, 315 studies (Supplementary file S1_Table S3) were selected for full-text review. After full-text screening, 100 studies were selected for data extraction (Supplementary file S1_Table S4), and 88 studies (Supplementary file S1_Table S5) met the criteria for inclusion in the descriptive synthesis. One additional dataset was derived from a study conducted in our laboratory whose data was complete and available at the time the database search was concluded (14 March 2024). This dataset was incorporated consistently with PRISMA 2020 guidance on the inclusion of unpublished data, and the study was subsequently published and is cited as Shrestha et al. (2025) [8], bringing the total eligible studies to 88. Of these 88 studies, 57 (Supplementary file S1_Table S6) met the criteria for systematic review, whereas 47 (Supplementary file S1_Table S7) and 35 (Supplementary file S1_Table S8) qualified for inclusion in the quantitative meta-analysis for source and serogroup.

**Figure 2:**
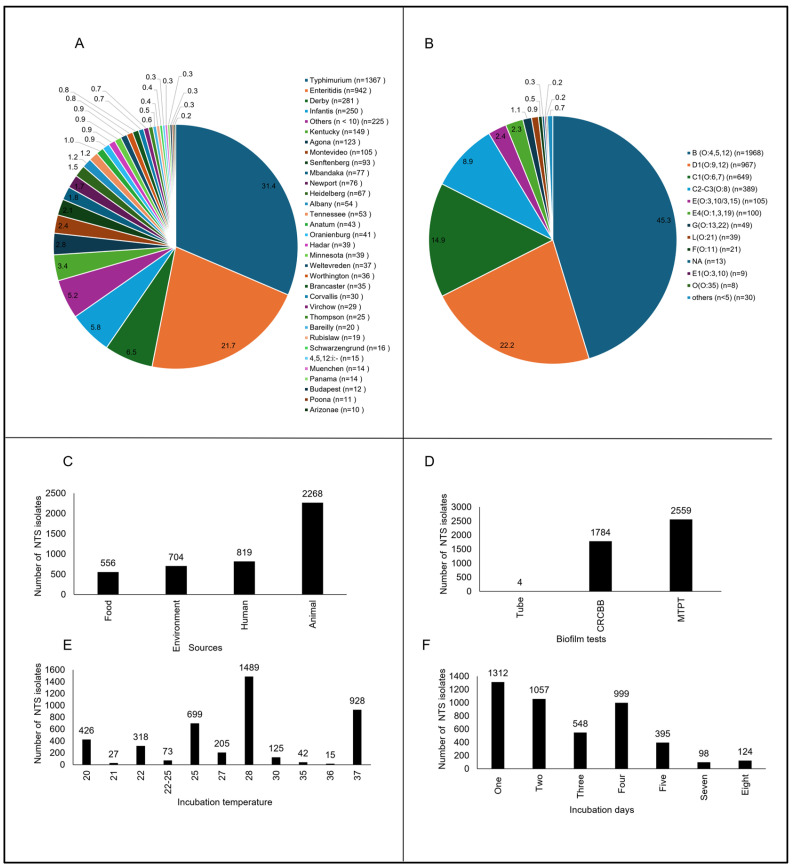
Descriptive synthesis of the biological and methodological structure of the systematic review dataset. (**A**) Distribution of non-typhoidal *Salmonella enterica* serotypes; (**B**) serogroups; (**C**) ecological source categories; (**D**) biofilm tests; (**E**) incubation temperatures; and (**F**) incubation durations used for biofilm assessment are represented in the full dataset (*n* = 88 studies).

The biological and methodological structure of the dataset from 88 studies included in the systematic review is summarized in Figure 2. Across the 88 studies, a total of 4347 NTS isolates representing 128 serotypes (Figure 2A) and 26 serogroups (Figure 2B) were evaluated for biofilm formation. Isolates were derived from multiple ecological sources, with the highest number of isolates originating from animals, followed by humans, the environment, and food (Figure 2C). The microtiter plate test was the most frequently used method for biofilm assessment, followed by the CRCBB and tube test (Figure 2D). Incubation temperatures ranged from 20 °C–37 °C, with 37 °C being the most used condition (Figure 2E), and incubation durations ranged from 1–8 days (Figure 2F). Together, these data show substantial heterogeneity in methodological approaches across studies, along with significant variance in inferences about biofilm phenotypes, thereby justifying the standardization of biofilm phenotypes before descriptive synthesis and meta-analyses.

### 3.2. Systematic Review

**Figure 3:**
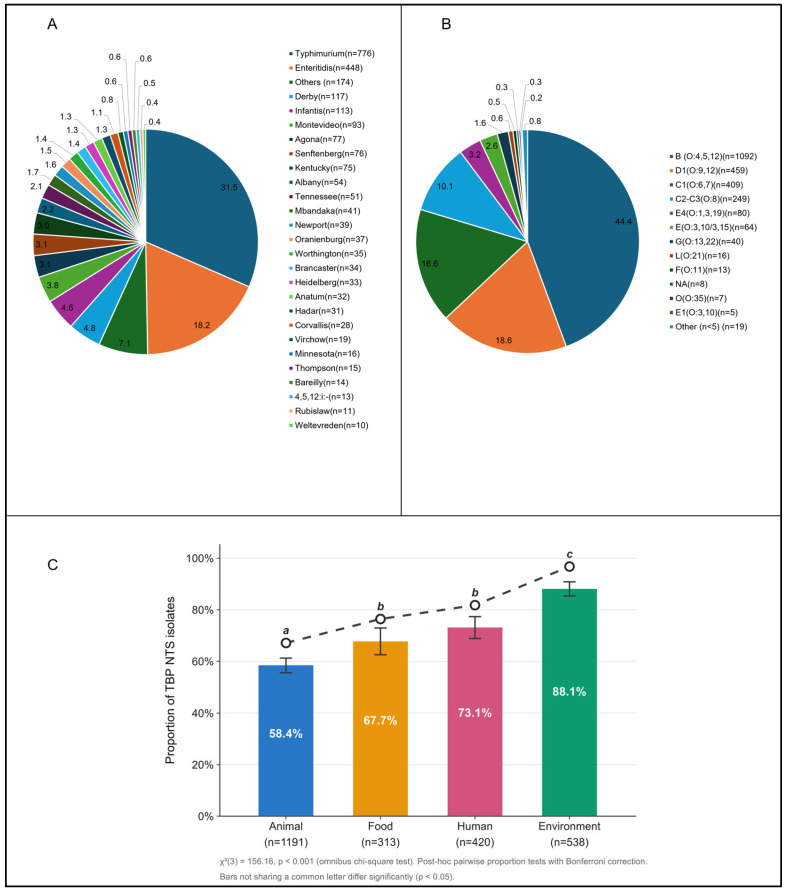
Plates (**A**,**B**) show the distribution of NTS serotypes and serogroups, respectively, among the studies (*n* = 57) that contain data describing TBP and TBN NTS isolates. Plate (**C**) shows the proportion of TBP NTS isolates from animal, food, human, and environmental sources across a subset of studies (*n* = 57). Bars represent the proportion of TBP isolates within each source category; error bars indicate 95% confidence intervals. Significance markers a, b, and c correspond to pairwise comparisons representing significant differences between each other (*p* < 0.001).

For the systematic review, the source of NTS isolation was grouped into four broad ecological source categories: animal, food, human, and environment, and for each source category, strong biofilm producers were considered TBP, and non-producers were considered TBN, as described in the methods. A total of 31 out of 88 studies without a TBP or a TBN category were excluded, resulting in a total of 57 studies for downstream analysis (Figure 3). These studies collectively contained 2462 NTS isolates representing 101 serotypes (Figure 3A) and 21 serogroups (Figure 3B), of which 1689 were TBP and 773 were TBN. The TBP proportional analysis showed a linear trend (Figure 3C), increasing in prevalence from animal sources (58.4%) to food sources (67.7%) to human sources (73.1%), with environmental isolates showing the highest prevalence (88.1%) (Supplementary file S2_Table S9). The *chi*-square test of homogeneity indicated a statistically significant difference in TBP proportion across the four source categories (χ^2^ = 201.08, *p* < 0.001). Pairwise comparisons using Bonferroni-adjusted two-proportion *z*-tests showed that all four source categories differed significantly from one another except food and human (Supplementary file S2_Table S10).

The source-attributed proportional analysis shows that animal reservoirs contain a nearly equal proportional distribution of TBP and TBN NTS, whereas food, humans, and environments outside of animal reservoir settings are enriched with TBP NTS (Figure 3). Environmental isolates showed the highest TBP prevalence, which is likely because these isolates originated from diverse niches, including farm environments, slaughterhouses, processing environments, and feed-related settings where NTS can be expected to be exposed to recurring stressors, including desiccation, nutrient limitation, temperature fluctuations, and sanitizer exposure, as discussed below. Food-associated isolates also showed markedly higher TBP prevalence than animal isolates, supporting the view that post-harvest processing may also impose selection pressure for enrichment of TBP, as the biofilm is known to afford protection against sanitation and promote persistence in the food chain [22,23]. Given the non-longitudinal nature of the datasets, these results do not demonstrate a direct epidemiological link from animal to food to humans; however, these results do show a biologically coherent enrichment pattern consistent with the idea that TBP strains are more likely to survive the transitions that shape the farm-to-fork continuum [24]. In practical terms, this trend strengthens the argument that biofilm formation deserves greater attention in both food safety surveillance and intervention design. Several extra-host stress conditions documented in the published literature are consistent with the observed enrichment of TBP NTS outside the animal reservoir. Desiccation and low water activity, prevalent in food-processing environments, have been shown to select for strongly adherent, biofilm-forming NTS subpopulations that can survive extended dry periods on equipment surfaces [25,26]. Repeated exposure to sanitizers and disinfectants at sub-lethal concentrations, a common feature of food-processing cycles, has been shown to promote biofilm formation and increase resistance to subsequent sanitation treatments [27,28]. Nutrient limitation and temperature fluctuations in environmental niches such as farm runoff, slaughterhouse drains, and processing facility surfaces further impose conditions that favor the persistence of matrix-encased biofilm-forming variants over planktonic cells [22,29]. These mechanisms collectively provide a biological framework for interpreting the source gradient observed in our meta-analysis, in which the progressive enrichment of TBP NTS from animal to food to environmental sources likely reflects the cumulative selective pressure imposed by these stressors across the farm-to-fork continuum.

### 3.3. Meta-Analysis of Biofilm Prevalence Across Source and Serogroup Categories

To improve statistical precision and minimize sparse subgroup estimates, observations were filtered so that only ecological source categories and serogroups represented by at least five independent studies and studies containing a minimum of 10 isolates per study-level observation were included in the quantitative meta-analysis, as reported previously [15]. A total of 47 and 35 unique studies were included in the source-level and serogroup-level meta-analyses, respectively.

A multilevel random-effects meta-analysis model was employed using the logit-transformed proportion of TBP NTS isolates as the primary effect size. Because multiple observations were frequently derived from the same publication, the study identifier was included as a random effect to account for within-study dependence. The baseline random-effects model yielded a predicted TBP proportion of 73.87% (95% CI: 58.40–85.06%) across the 47 studies included in the meta-analysis. Substantial between-study heterogeneity was observed (τ^2^ = 5.12; Q (df = 54) = 434.98, *p* < 0.0001), indicating considerable variability across studies. In the meta-analysis with serogroup as a moderator (35 studies), the baseline pooled TBP proportion was 72.99% (95% CI: 53.66–86.31%), with similarly high heterogeneity (τ^2^ = 5.68; Q (df = 49) = 375.488, *p* < 0.0001).

#### 3.3.1. Source-Level Meta-Analysis

To investigate whether the ecological source explained some of this heterogeneity, a multilevel mixed-effects meta-regression model was constructed with the source of isolation as the moderator. Adding the source significantly improved model fit compared with the baseline model, and the omnibus moderator test confirmed that the source collectively explained a statistically significant portion of between-study variability (QM (df = 3) = 17.07, *p* = 0.0007). Although the amount of variance explained was modest (R^2^ = 3.6%; τ^2^ = 4.96), the direction of the source effects was clear and biologically informative.

**Figure 4:**
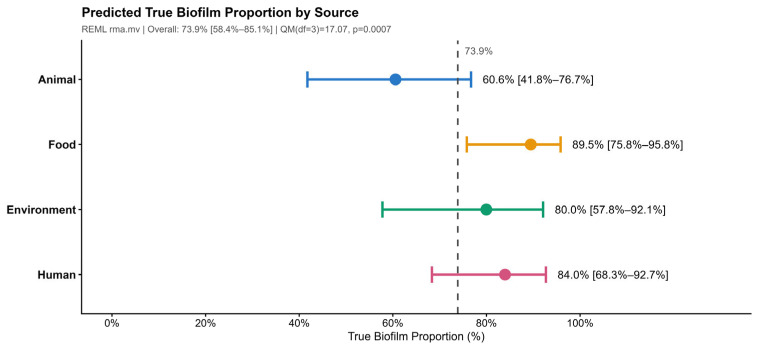
Summary plot of predicted true-biofilm positive (TBP) proportions by source of isolation derived from the REML multilevel meta-regression model (rma.mv). Points represent back-transformed predicted proportions for each source, with horizontal error bars indicating 95% confidence intervals. The dashed vertical line indicates the overall pooled proportion from the baseline model.

The meta-regression model indicated that the predicted proportion of TBP NTS isolates was lowest among animal-derived sources (Figure 4; Supplementary file S2_Table S11). Similarly, pairwise comparison showed that the predicted proportions of TBP isolates from food (*p* = 0.0002) and human (*p* = 0.0005) sources were significantly higher than those from animal sources (Supplementary file S2_Table S12). No significant difference was observed between food and human sources, suggesting that once TBP NTS crosses the selection pressure at the food-processing intersection, the relative prevalence of TBP isolates may remain comparable between the food and human endpoints of the food chain.

Environmental isolates also showed numerically higher predicted TBP proportions than animal isolates, although this difference did not reach statistical significance (*p* = 0.075). This apparent discrepancy with the raw proportional analysis, in which all four source categories differed significantly, reflects fundamental differences in how the two approaches weight evidence. The raw analysis pools all isolates equally across studies without accounting for study-level dependence, giving disproportionate influence to larger studies. The multilevel meta-regression weights study-level estimates by inverse variance and accounts for within-study dependence, which is statistically more rigorous but also more sensitive to the number and heterogeneity of available studies per category. The environmental category had the fewest studies (*n* = 9) and the highest within-category heterogeneity, reducing statistical power for this specific contrast. However, the direction of the environmental effect is consistent across both analyses; environmental isolates showed the highest TBP prevalence numerically in both approaches, suggesting that non-significance reflects a power limitation rather than a true null ecological difference.

The combination of the descriptive source gradient and the source-level meta-regression suggests that TBP NTS are not randomly distributed across ecological sources. Rather, they appear to be relatively enriched outside the primary animal reservoir in settings where environmental stress and sanitation pressure may favor strains able to persist through attachment and matrix formation [26,28]. In particular, the elevation of TBP prevalence in food-associated sources is epidemiologically important because it is consistent with a scenario in which TBP NTS are more likely to survive post-harvest extra-host stress conditions (discussed in detail in Section 3.2) [11,25,27,30]. Such stressful conditions may preferentially enrich strongly adherent, biofilm-forming NTS subpopulations in foods that may remain viable for human exposure [28,29]. This may explain the lack of significant differences in the predicted proportion of TBP NTS isolates among food and human sources.

**Figure 5:**
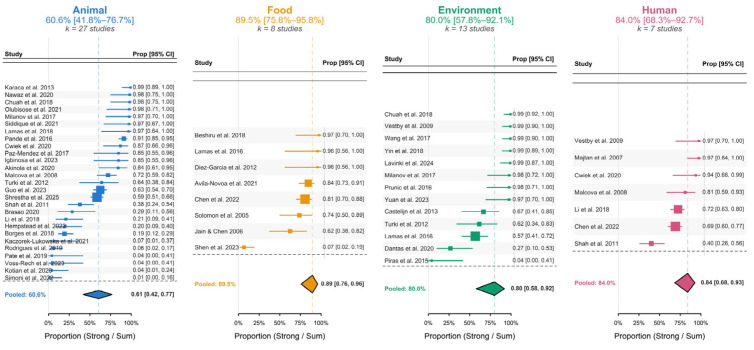
Forest plots of source-specific pooled proportions of TBP NTS isolates. Separate panels are shown for animals, food, environment, and human sources. Squares represent study-specific proportion estimates on the proportion scale (0–100%), with horizontal lines indicating 95% confidence intervals. Square size is proportional to study weight. The diamond at the bottom of each panel represents the back-transformed pooled logit proportion for that source subgroup derived from the multilevel random-effects model, with the center of the diamond indicating the pooled estimate and the diamond width indicating the 95% confidence interval of that pooled estimate.

The source-stratified forest plots (Figure 5) show that, despite variability across individual studies, the pooled TBP proportion for animal-derived isolates is lower relative to the pooled estimates for food, human, and environmental sources. While several individual animal studies report high TBP prevalence, the overall subgroup estimate, accounting for study-level variability and weighting, shifts lower compared to sources outside of animal reservoirs. This relative difference is consistent with the meta-regression results and supports the interpretation that TBP NTS are comparatively enriched outside the primary animal reservoir. The higher pooled estimates observed for food- and human-associated isolates are therefore consistent with the hypothesis that strains capable of strong biofilm formation are more likely to persist under extra-host conditions and be recovered in downstream stages of the food-production continuum [29].

#### 3.3.2. Serogroup-Level Meta-Analysis

**Figure 6:**
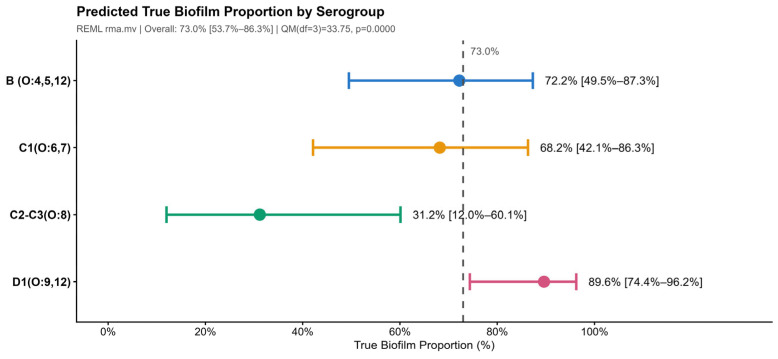
Summary plot of predicted TBP proportions by serogroup derived from the REML multilevel meta-regression model (rma.mv). Points represent back-transformed predicted proportions for each serogroup, with horizontal error bars indicating 95% confidence intervals. The dashed vertical line indicates the overall pooled proportion from the baseline model.

A secondary meta-analysis was performed using 35 studies (details in the Supplementary file S1_Table S8) (Figure 6) meeting the predefined criteria for serogroup as moderator (QM (df = 3) = 33.75, *p* < 0.0001). Serogroup D1 (O:9,12) showed the highest predicted TBP proportion (89.62%, 95% CI: 74.37–96.25%), followed by serogroup B (O:4,5,12), serogroup C1 (O:6,7), and serogroup C2–C3 (O:8), which showed the lowest predicted proportion (Supplementary file S2_Table S13). Pairwise comparisons showed that D1 was significantly higher than B, C1, and C2–C3, whereas C2–C3 was significantly lower than all other major serogroups (Supplementary file S2_Table S14). While these serogroup results are interesting, the dataset was not balanced across serogroups or serotypes within serogroups. For example, Kentucky and Albany were highly represented within serogroup C2–C3, whereas serogroup D1 included Enteritidis, Blegdam, Berta, and Panama. Isolates belonging to serotype Kentucky have been reported to be weak biofilm producers and Enteritidis as strong biofilm producers by a few other studies, too [30,31,32]. Wide confidence intervals for serogroup C2-C3 (predicted proportion 12–60%) reflect both limited study representation and substantial within-serogroup heterogeneity driven by the serotype composition, particularly the dominance of *S. kentucky*, a serotype frequently reported as a weak biofilm producer [31,32,33]. Serogroup-level estimates with wide confidence intervals should therefore be interpreted as exploratory indicators rather than precise prevalence values, as inference at this level is constrained by dataset sparsity and serotypic heterogeneity within serogroups. Importantly, the consistently high prevalence of BP isolates across most serogroups indicates that biofilm formation is not restricted to a limited subset of NTS but rather represents a broadly distributed adaptive phenotype. Study-level TBP prevalence, stratified by serogroup, is shown as a forest plot across 35 studies (Supplementary file S3_Figure S1).

#### 3.3.3. Heterogeneity Summary and Publication Bias

The baseline model revealed substantial between-study heterogeneity for both source-level and serogroup-level analyses (Table 1). The source of isolation explained only a modest fraction of the between-study variance, while serogroup, despite being a statistically significant moderator, did not meaningfully reduce the residual variance component (Supplementary file S2_Table S15). This persistent residual heterogeneity indicates that biofilm-forming prevalence is influenced by additional unmeasured factors, likely including serotype- or strain-level genetic variation, assay-specific methodological differences, and ecological or geographic differences not fully captured in the available literature.

The multilevel REML random-effects model was selected because it explicitly models between-study variance rather than assuming homogeneity, making it the appropriate analytic framework for prevalence meta-analyses with high heterogeneity (I^2^) [16,34]. The REML estimator is preferred for variance component estimation in mixed models because it produces unbiased estimates of tau^2^ and is robust to the choice of starting values. Nine potential moderators were screened in univariable analyses (Section 2.9); only the source of isolation and biofilm test method reached significance in the omnibus moderator test (QM *p* < 0.05) and were retained in the best-fitting multivariable model (Model 2). The remaining moderators, incubation temperature, incubation duration, year of publication, and individual serogroup indicators did not meaningfully reduce residual heterogeneity and were excluded on parsimony grounds, as confirmed by AIC and BIC comparisons. Substantial residual heterogeneity persisted in Model 2 (I^2^ = 95.5%), indicating that unmeasured factors, including strain-level genetic diversity, geographic variation, and assay-specific procedural details not consistently reported across studies, account for a large fraction of between-study variability. Readers should therefore interpret the pooled prevalence estimates as central tendencies across a highly heterogeneous body of literature rather than precise point estimates and the source-level contrasts as directional signals supported by formal moderator testing rather than definitive prevalence values.

Funnel plots and trim-and-fill analyses are provided in Supplementary file S3_Figure S2 and Supplementary file S3_Figure S3, respectively, for source-aggregated data (containing 47 studies) and serogroup-aggregated data (containing 35 studies). However, trim and fill adjustment was not used as the primary bias adjustment because it performs poorly under high heterogeneity (I^2^ > 95%) and for proportion meta-analyses, where funnel-plot asymmetry reflects genuine between-study differences rather than missing studies [34,35]. No significant publication bias was detected for either source-level or serogroup-level analyses based on Egger’s regression test and Begg’s rank correlation test (Supplementary file S2_Table S16). The publication bias analyses were conducted using a standard univariate random-effects model (.rma), which yielded a pooled estimate of 75.62%, compared with the 73.87% reported in Section 3.3 from the multilevel model (rma.mv); the small difference reflects the two models’ distinct structures rather than an analytical inconsistency. The trim-and-fill method imputed 8 potentially missing studies for the source-level analysis, shifting the adjusted pooled estimate from 75.62–65.66%. However, the trim-and-fill method is known to perform poorly under high heterogeneity (I^2^ > 95%), where funnel plot asymmetry reflects genuine between-study variation rather than missing studies [35,36]. The primary inferential conclusions of this study rest on source-level meta-regression contrasts rather than the absolute pooled estimate, and the direction and relative ordering of source-level differences remain unaffected by this adjustment.

#### 3.3.4. Univariable, Multivariable Meta-Regression, and Best Model Selection

Univariable mixed-effects meta-regression, including nine moderators one at a time, showed source and biofilm test as the significant moderators based on the omnibus test of moderators (QM) *p*-value < 0.05 (Supplementary file S4_Table S17). Further, we did multivariable meta-regression including moderators: incubation temperature, incubation days, source, incubation tests, serogroup D1 (O:9,12), serogroup C1 (O:6,7), serogroup C2–C3 (O:8), and serogroup B (O: 4,5,12), leading to a full model (Model 1). A reduced multivariable model (Model 2) was then fitted using only the moderators significant in the univariable analyses: source of isolation and biofilm test. Based on the lower between-study variance (τ^2^) and Higgins’ I^2^ [34] but higher meta-analytic R^2^, model 2 (with source and test moderator) was selected (Supplementary file S4_Table S18). Further, an ANOVA comparison of the two models determined no significant differences in the AIC, BIC, and AICc values. The full model (model 1) was not improved over the reduced source-and-test method model 2 (likelihood ratio test χ^2^ = 3.30, df = 7, *p* = 0.86), and all information criteria favored the reduced model (AIC 248.9 vs. 259.6; BIC 261.0 vs. 285.7; AIC 250.7 vs. 268.5, based on the ML method). The model 2 containing source and test was therefore retained as the best-fitting and most parsimonious, with τ^2^ = 4.6 and I^2^ = 95.5%, and reduced heterogeneity by 10.1%. The full multivariable coefficient of model 2 is provided as Supplementary file S4_Table S19. In the best-fitting multivariable model (Model 2; source and biofilm test as moderators), food-associated isolates showed significantly higher odds of TBP relative to animal-derived isolates (OR = 5.07; 95% CI: 2.07–12.41; *p* = 0.0004), as did human-associated isolates (OR = 3.22; 95% CI: 1.62–6.43; *p* = 0.0009). Environmental isolates showed higher but non-significant odds relative to animal sources (OR = 2.47; 95% CI: 0.87–7.03; *p* = 0.090). The microtiter plate test (MTPT) detected marginally lower odds of TBP than the CRCBB test (OR = 0.26; 95% CI: 0.07–1.01; *p* = 0.052), an association that approached but did not reach statistical significance. Since CRCBB is a qualitative test and MTPT is a quantitative-based biofilm detection method, CRCBB may have higher confidence in predicting TBP NTS. Full model coefficients are provided in Supplementary File S4_Table S20.

#### 3.3.5. Conclusions

This systematic review and meta-analysis support the hypothesis that ecological sources influence TBP prevalence in NTS. Across both descriptive and meta-analytic approaches, animal-derived isolates consistently showed the lowest prevalence of TBP isolates, whereas food- and human-associated isolates showed significantly higher prevalence. Environmental isolates also showed high prevalence, consistent with the view that extra-host niches impose conditions favorable to the survival of TBP strains.

Taken together, these results suggest that biofilm formation may contribute to an enrichment pathway across the farm-to-fork continuum in which strong biofilm-forming NTS are more likely than non-biofilm producers to persist outside the animal reservoir, survive food production and processing conditions, and remain available for human exposure. While this study does not establish direct transmission or causation, it does provide a strong epidemiologically meaningful signal that such a linkage likely exists and warrants follow-up investigations in longitudinal studies. Future studies should account for serovar distribution across source categories, as the overrepresentation of weak biofilm-forming serotypes such as *S*. Kentucky in certain source categories may partially confound source-level prevalence estimates.

The findings also have practical relevance. If TBP NTS are preferentially enriched as strains move through extra-host production environments, then biofilm phenotype may serve as a useful adjunct marker of persistence and public health risk in surveillance and outbreak investigations. This has implications for both pre-harvest control, where reducing biofilm-forming NTS in animal production could lower the pool available to enter the food chain, and post-harvest control, where identifying biofilm-forming strains may help justify enhanced sanitation and targeted intervention in processing environments. For these reasons, incorporating biofilm phenotype into future surveillance and intervention research may improve risk prediction and ultimately reduce the burden of human NTS infection.

Several limitations of this systematic review and meta-analysis should be acknowledged. First, this review was restricted to English-language publications, which may have introduced language bias by excluding relevant studies published in other languages. Future updates to this review should attempt to incorporate translated or multilingual records. Second, this systematic review was retrospectively registered with OSF Registries during peer review (10.17605/OSF.IO/2694M), reducing protection against selective outcome reporting. Third, substantial residual heterogeneity persisted after moderator inclusion (I^2^ approximately 95.5%), indicating that unmeasured factors, including strain-level genetic variation, geographic differences, and assay-specific procedural details, account for a large fraction of between-study variability, and pooled estimates should be interpreted as central tendencies rather than precise prevalence values. Fourth, the serotype composition within serogroup categories was not balanced, particularly for serogroup C2–C3, where *S*. Kentucky dominated; this may have influenced serogroup-level pooled estimates and limited the generalizability of serogroup-level inferences. Fifth, restriction to studies with at least five independent observations per source or serogroup category, while necessary for statistical stability, excluded smaller studies that may have captured important biological variation. Finally, the cross-sectional and non-longitudinal nature of the included studies precludes causal inference regarding the enrichment of biofilm-forming NTS across the farm-to-fork continuum; the observed source gradient is consistent with but does not demonstrate direct transmission or selection pressure.

## Figures and Tables

**Table 1:** Heterogeneity summary for baseline and moderator models.

Analysis	Model	tau^2^	Q (df)	*p*	R^2^
Source	Baseline	5.12	434.98 (54)	<0.0001	—
Source	+Source	4.96	QE = 411.23 (51)	<0.0001	3.15%
Serogroup	Baseline	5.69	375.49 (49)	<0.0001	—
Serogroup	+Serogroup	7.15	QE = 332.06 (46)	<0.0001	0.0%

## Data Availability

Data supporting reported results can be found in the Supplementary Files.

## Supplementary Materials

The following supporting information can be downloaded at: https://www.mdpi.com/article/10.3390/microorganisms14071584/s1. Supplementary file S1: Table S1 Compilation of articles related to biofilm and Salmonella from Web of Science, Scopus, and Pub med; Table S2 Deduplicaiton of studies; Table S3 A total of 315 studies were selected based on the abstract reading; Table S4 A total of 100 studies selected based on full paper reading and data extraction; Table S5 A total of 88 studies were selected after removing all the undefined and unknown sources; Table S6 A total of 57 studies selected for the systematic review; Table S7 A total of 47 studies used for source-based meta-analysis; Table S8 A total of 35 studies used for serogroup-based meta-analysis, Supplementary file S2: Table S9 Descriptive statistics for the proportion of true biofilm-positive by ecological sources (*n* = 57 studies); Table S10 Pairwise comparisons of the true biofilm-positive (TBP) proportions (Bonferroni Corrected); Table S11 Predicted proportion of TBP based on source Meta-analysis (*n* = 47 studies); Table S12 Pairwise comparisons of TBP predicted proportion between source categories; Table S13 Predicted proportion of TBP based on serogroup Meta-analysis (*n* = 35 studies); Table S14 Pairwise comparisons of TBP predicted proportion between source categories; Table S15 Heterogeneity and model fit statistics for source-level and serogroup-level meta-analyses; Table S16 Egger’s and Begg’s tests for publication bias summary—source and serogroup analyses, Supplementary file S3: Figure S1. Forest plots of pooled TBP proportions by serogroup; Figure S2. Publication bias assessment—source-level meta-analysis (*k* = 47 studies); Figure S3. Publication bias assessment—serogroup-level meta-analysis (*k* = 35 studies), Supplementary file S4: Table S17 Univariable mixed-effects meta-regression of moderators of TBP in NTS (*k* = 47 studies, 55 observations).; Table S18 Model comparison full model (model1) and reduced model (model2)_REML method; Table S19 Multivariate coefficients of full model; Table S20 Full model coefficients for the best-fitting multivariable meta-regression model (Model 2) containing source of isolation and biofilm test method as moderators, and Supplementary file S5 Systematic review and meta-analysis of biofilm-forming non-typhoidal salmonella enterica across the farm-to-fork continuum.

## Abbreviations

The following abbreviations are used in this manuscript:

AIC	Akaike Information Criterion
ANOVA	Analysis of Variance
BIC	Bayesian Information Criterion
CI	Confidence Interval
CRCBB	Congo Red Coomassie Brilliant Blue (Congo red and Coomassie brilliant blue dyes added in LB agar, no salt to make CRCBB agar)
DF	Degrees of Freedom
NTS	Non-Typhoidal *Salmonella*
MTPT	Microtiter Plate Test
PRISMA	Preferred Reporting Items for Systematic Reviews and Meta-Analyses
QM	Q-statistic for Moderators (omnibus test of moderators)
RDAR	Red, Dry, and Rough (colony morphotype)
REML	Restricted Maximum Likelihood
SAW	Smooth and White (colony morphotype)
TBP	True Biofilm Positive (Strong Biofilm Positive)
TBN	True Biofilm Negative (Biofilm Negative)
