# Supplementary material for: Prevalence of Biofilm-Forming Non-Typhoidal Salmonella Across the Farm-to-Fork Continuum: A Systematic Review and Meta-Analysis
Source: Microorganisms. 2026 Jul 20;14(7):1584. doi: 10.3390/microorganisms14071584 (PMC13414013; doi:10.3390/microorganisms14071584)
Supplement: Supplementary file 1 [file microorganisms-14-01584-s001.zip › Supplementary file S3.pdf]

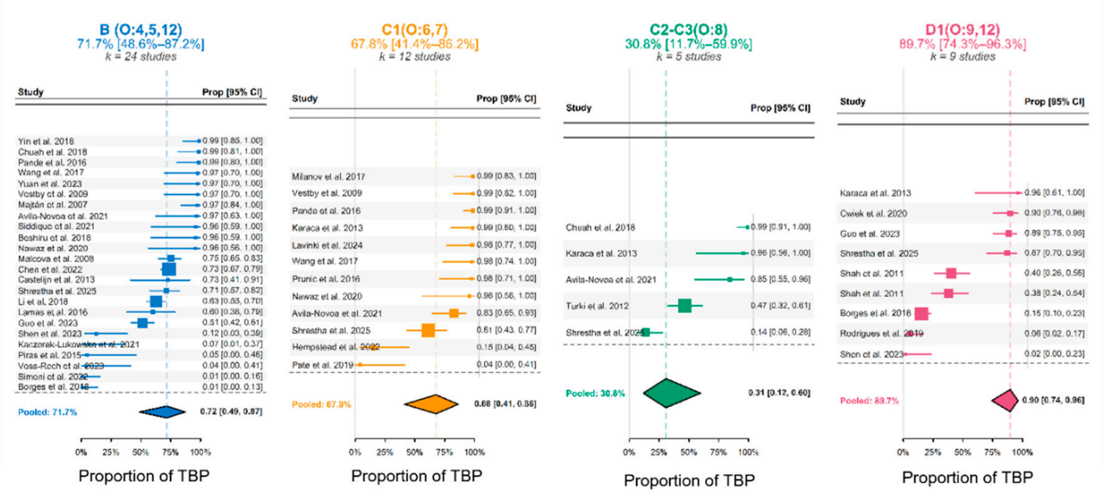

Figure S1. Forest plots of pooled TBP proportions by serogroup. Four serogroups with at least five studies and a minimum of 10 isolates per observation are displayed: B (O:4,5,12), C1 (O:6,7), C2–C3 (O:8), and D1 (O:9,12). Individual study estimates (squares) are plotted on the proportion scale (0–100%), with horizontal lines representing 95% confidence intervals. Square size is proportional to study weight. The pooled estimate for each serogroup is represented by a colored diamond at the bottom of each panel. Analysis was performed using a multilevel random-effects model (rma.mv, REML estimation) with study identifier as a random effect. Effect sizes were logit-transformed proportions and back-transformed for display.

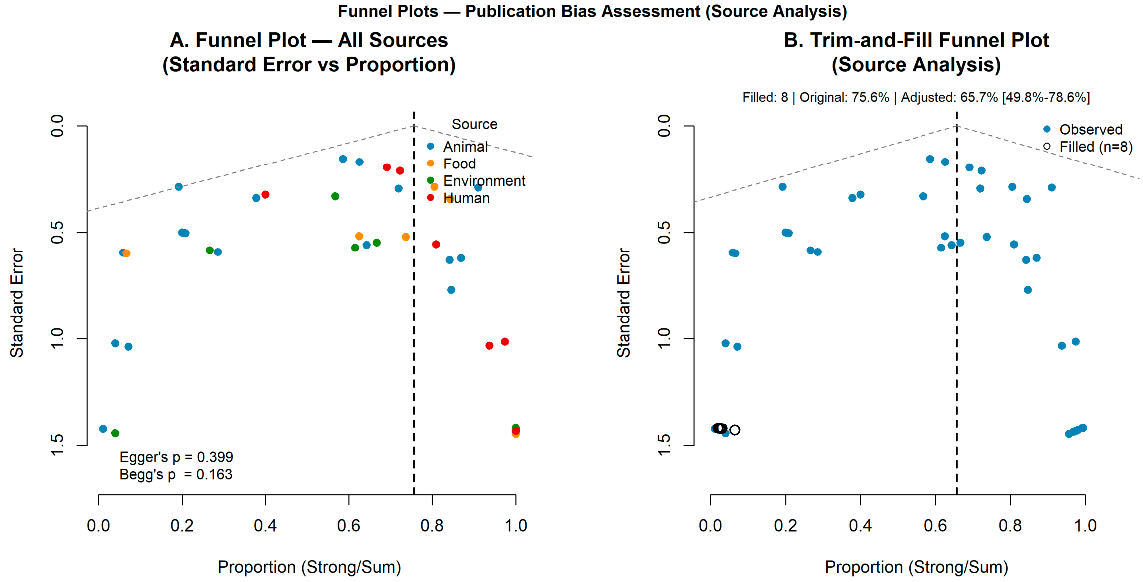

Figure S2. Publication bias assessment — source-level meta-analysis (k = 47 studies). (A) Standard funnel plot of strong biofilm proportion vs. standard error, color-coded by source (Animal=blue, Food=orange, Environment=green, Human=red). Dashed vertical line = pooled estimate (75.6%); diagonal dashed lines = pseudo-95% CI. No significant asymmetry was detected (Egger's p = 0.399; Begg's $\tau = 0.13$, p = 0.163). (B) Trim-and-fill funnel plot; open circles indicate 8 imputed studies. Adjusted pooled estimate: 65.7% [49.8%–78.6%] vs. original 75.6% [62.4%–85.3%]. Funnel plot

asymmetry in meta-analyses with high between-study heterogeneity ($I^2 > 95\%$) primarily reflects genuine variation across studies rather than missing studies due to publication bias and should not be interpreted as definitive evidence of publication bias in isolation from formal statistical tests. Egger's regression test and Begg's rank correlation test were used as formal assessments of publication bias, and results are reported in Supplementary File S2 Table S8.

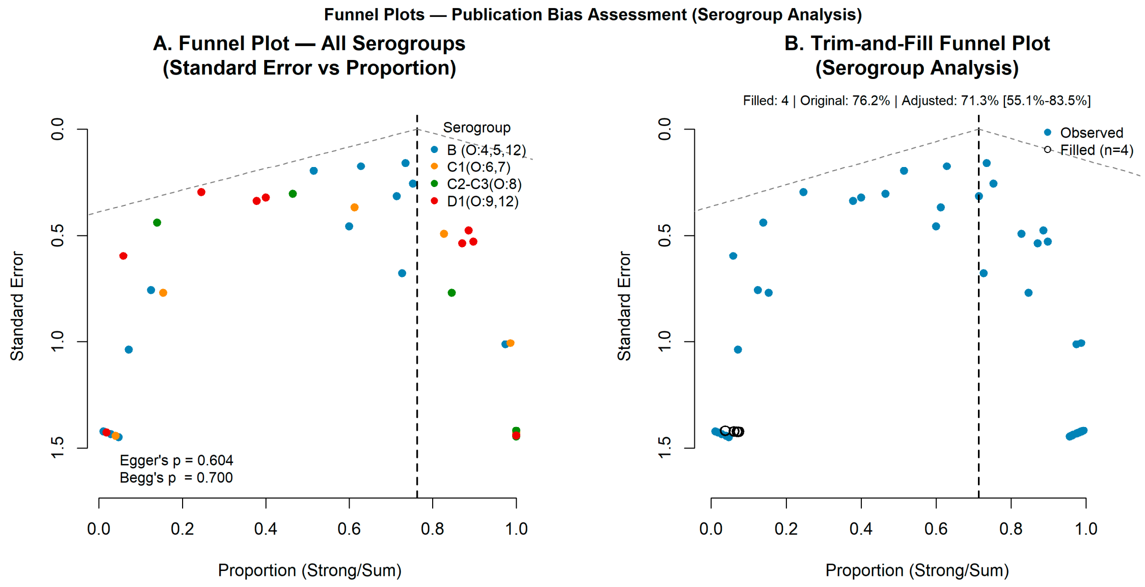

Figure S3. Publication bias assessment — serogroup-level meta-analysis (k = 35 studies). (A) Standard funnel plot of strong biofilm proportion vs. standard error, color-coded by source (Animal=blue, Food=orange, Environment=green, Human=red). Dashed vertical line = pooled estimate (75.4%); diagonal dashed lines = pseudo-95% CI. No significant asymmetry was detected (Egger's $p = 0.604$; Begg's $\tau = -0.038$, $p = 0.700$). (B) Trim-and-fill funnel plot; open circles indicate 4 imputed studies. Adjusted pooled estimate: 71.3% [55.1%–83.5%] vs. original 76.2% [61.2%–86.7%]. Funnel plot asymmetry in meta-analyses with high between-study heterogeneity ($I^2 > 95\%$) primarily reflects genuine variation across studies rather than missing studies due to publication bias, and should not be interpreted as definitive evidence of publication bias in isolation from formal statistical tests. Egger's regression test and Begg's rank correlation test were used as formal assessments of publication bias, and results are reported in Supplementary File S2 Table S8.
