# Supplementary material for: Prevalence of Biofilm-Forming Non-Typhoidal Salmonella Across the Farm-to-Fork Continuum: A Systematic Review and Meta-Analysis
Source: Microorganisms. 2026 Jul 20;14(7):1584. doi: 10.3390/microorganisms14071584 (PMC13414013; doi:10.3390/microorganisms14071584)
Supplement: Supplementary file 1 [file microorganisms-14-01584-s001.zip › Supplementary File S5.pdf]

PRISMA 2020 Checklist

Systematic Review and Meta-Analysis of Biofilm-Forming Non-Typhoidal Salmonella enterica across the Farm-to-Fork Continuum

Adapted from: Page MJ, McKenzie JE, Bossuyt PM, et al. The PRISMA 2020 statement: an updated guideline for reporting systematic reviews. BMJ 2021;372:n71. Distributed under CC BY 4.0.

Item #	Checklist item	Status
**TITLE**		
1	Identify the report as a systematic review.	**OK**
**ABSTRACT**		
2	Structured summary including background, objectives, eligibility criteria, information sources, risk-of-bias appraisal, synthesis methods, results, limitations, funding, and registration.	**OK**
**INTRODUCTION**		
3	Rationale: Describe the rationale for the review in the context of existing knowledge.	**OK**
4	Objectives: provide an explicit statement of the objective(s) or question(s) the review addresses.	**OK**
**METHODS**		
5	Eligibility criteria: specify the inclusion and exclusion criteria for the review and how studies were grouped for synthesis.	**OK**
6	Information sources: specify all databases, registers, websites, organizations, reference lists, and other sources searched, with the date of last search for each.	**OK**
7	Search strategy: present the full search strategy for at least one database, including filters and limits used.	**OK**
8	Selection process: specify the methods used to decide whether a study met inclusion criteria, including number of reviewers, whether independently, and use of automation tools.	**OK**
9	Data collection process: specify methods used to collect data, including number of reviewers, whether independently, any process for obtaining data from study authors, and use of automation tools.	**OK**
10a	Data items: list and define all outcomes for which data were sought.	**OK**
10b	Data items: list and define all other variables for which data were sought (e.g., participant/study characteristics, funding sources) and any assumptions made about missing/unclear information.	**OK**
11	Study risk of bias assessment: specify the methods used to assess risk of bias in included studies, including number of reviewers, whether independently, and any tools used.	**OK**
12	Effect measures: specify for each outcome the effect measure(s) used in the synthesis or presentation of results.	**OK**
13a	Synthesis methods: describe the process used to decide which studies were eligible for each synthesis.	**OK**
13b	Synthesis methods: describe methods for preparing data for presentation or synthesis (e.g., handling of missing summary statistics, data conversions).	**OK**
13c	Synthesis methods: describe methods for tabulating or visually displaying results of individual studies and syntheses.	**OK**
13d	Synthesis methods: describe methods used to synthesize results (e.g., meta-analysis model) and justify the choice.	**OK**

Item #	Checklist item	Status
13e	Synthesis methods: describe methods used to explore possible causes of heterogeneity among study results (e.g., subgroup analysis, meta-regression).	**OK**
13f	Synthesis methods: describe sensitivity analyses conducted to assess robustness of synthesized results.	**OK**
14	Reporting bias assessment: describe methods used to assess risk of bias due to missing results in a synthesis.	**OK**
15	Certainty assessment: describe methods used to assess certainty (or confidence) in the body of evidence for an outcome.	**OK**
**RESULTS**		
16a	Study selection: describe results of the search and selection process, from records identified to studies included, ideally with a flow diagram.	**OK**
16b	Study selection: cite studies that might appear eligible but were excluded, and explain why.	**OK**
17	Study characteristics: cite each included study and present its characteristics.	**OK**
18	Risk of bias in studies: present assessments of risk of bias for each included study.	**OK**
19	Results of individual studies: present, for each study, summary statistics for each outcome and, if synthesized, a forest plot.	**OK**
20a	Results of syntheses: briefly summarize characteristics and risk of bias of studies contributing to each synthesis.	**OK**
20b	Results of syntheses: present results of all statistical syntheses, including effect estimates and precision (e.g., confidence/credible intervals) and measures of heterogeneity.	**OK**
20c	Results of syntheses: present results of investigations of heterogeneity (e.g., subgroup analysis, meta-regression).	**OK**
20d	Results of syntheses: present results of sensitivity analyses.	**OK**
21	Reporting biases: present assessment of risk of bias due to missing results for each synthesis.	**OK**
22	Certainty of evidence: present assessment of certainty in the body of evidence for each outcome.	**OK**
**DISCUSSION**		
23a	Discussion: provide a general interpretation of results in the context of other evidence.	**OK**
23b	Discussion: discuss limitations of the included studies/processes used to gather evidence.	**OK**
23c	Discussion: discuss limitations of the review processes used.	**OK**
23d	Discussion: discuss implications of the results for practice, policy, and future research.	**OK**
**OTHER INFORMATION**		
24a	Registration and protocol: provide the registration name and number, or state that the review was not registered.	**OK**
24b	Registration and protocol: indicate where the review protocol can be accessed, or state that a protocol was not prepared.	**OK**
24c	Registration and protocol: describe amendments to information provided at registration or in the protocol.	**OK**

Item #	Checklist item	Status
25	Support: describe sources of financial or non-financial support for the review, and the role of funders/sponsors.	**OK**
26	Competing interests: declare any competing interests of review authors.	**OK**
27	Availability of data, code, and other materials: report which materials are publicly available and where they can be found.	**OK**
